# QSAR Model for Predicting the Cannabinoid Receptor 1 Binding Affinity and Dependence Potential of Synthetic Cannabinoids

**DOI:** 10.3390/molecules25246057

**Published:** 2020-12-21

**Authors:** Wonyoung Lee, So-Jung Park, Ji-Young Hwang, Kwang-Hyun Hur, Yong Sup Lee, Jongmin Kim, Xiaodi Zhao, Aekyung Park, Kyung Hoon Min, Choon-Gon Jang, Hyun-Ju Park

**Affiliations:** 1 School of Pharmacy, Sungkyunkwan University, Suwon 16419, Korea; wonyoung1007@naver.com (W.L.); sojung1024@gmail.com (S.-J.P.); innetjy@hotmail.com (J.-Y.H.); khh508@naver.com (K.-H.H.); zhaoxiaodi1019@gmail.com (X.Z.); 2 Department of Pharmacy, College of Pharmacy, Kyung Hee University, Seoul 02447, Korea; kyslee@khu.ac.kr; 3 College of Pharmacy, Chung-Ang University, Seoul 06974, Korea; drugfriend2@gmail.com (J.K.); khmin@cau.ac.kr (K.H.M.); 4 College of Pharmacy, Sunchon National University, Suncheon 57922, Korea; parkak11@scnu.ac.kr

**Keywords:** cannabinoid receptor 1, synthetic cannabinoids, quantitative structure-activity relationship, multiple linear regression, partial least squares regression, dependence and abuse potential

## Abstract

In recent years, there have been frequent reports on the adverse effects of synthetic cannabinoid (SC) abuse. SCs cause psychoactive effects, similar to those caused by marijuana, by binding and activating cannabinoid receptor 1 (CB1R) in the central nervous system. The aim of this study was to establish a reliable quantitative structure–activity relationship (QSAR) model to correlate the structures and physicochemical properties of various SCs with their CB1R-binding affinities. We prepared tetrahydrocannabinol (THC) and 14 SCs and their derivatives (naphthoylindoles, naphthoylnaphthalenes, benzoylindoles, and cyclohexylphenols) and determined their binding affinity to CB1R, which is known as a dependence-related target. We calculated the molecular descriptors for dataset compounds using an R/CDK (R package integrated with CDK, version 3.5.0) toolkit to build QSAR regression models. These models were established, and statistical evaluations were performed using the mlr and plsr packages in R software. The most reliable QSAR model was obtained from the partial least squares regression method via Y-randomization test and external validation. This model can be applied in vivo to predict the addictive properties of illicit new SCs. Using a limited number of dataset compounds and our own experimental activity data, we built a QSAR model for SCs with good predictability. This QSAR modeling approach provides a novel strategy for establishing an efficient tool to predict the abuse potential of various SCs and to control their illicit use.

## 1. Introduction

The quick and worldwide distribution of drugs to the general public, including young adults, via the online market has led to the emergence of drug abuse and drug addiction as crucial social issues. According to the United Nations Office on Drugs and Crime World Drug Report 2020, the worldwide estimated annual incidence of illicit drug use is the highest for cannabis, since the number of cannabis users was about 192 million in 2018 which is 3.9% of the global adult population aged 15–64 [1]. In terms of global substance dependence, cannabis and opioids are associated with the most common illicit drug dependence, accounting for 19.8 and 16.8 million cases, respectively, in 2015 [2]. Recently, a study on the correlation between prenatal exposure to cannabis and child neurodevelopment was conducted by the Ottawa Hospital Research Institute in Canada. The results showed that women who used cannabis during pregnancy were 1.5 times more likely to give birth to a child with autism than women who did not use cannabis [3]. The abuse of cannabis and synthetic cannabinoids (SCs) is associated with various harmful health effects and even death.

**Figure 1:**
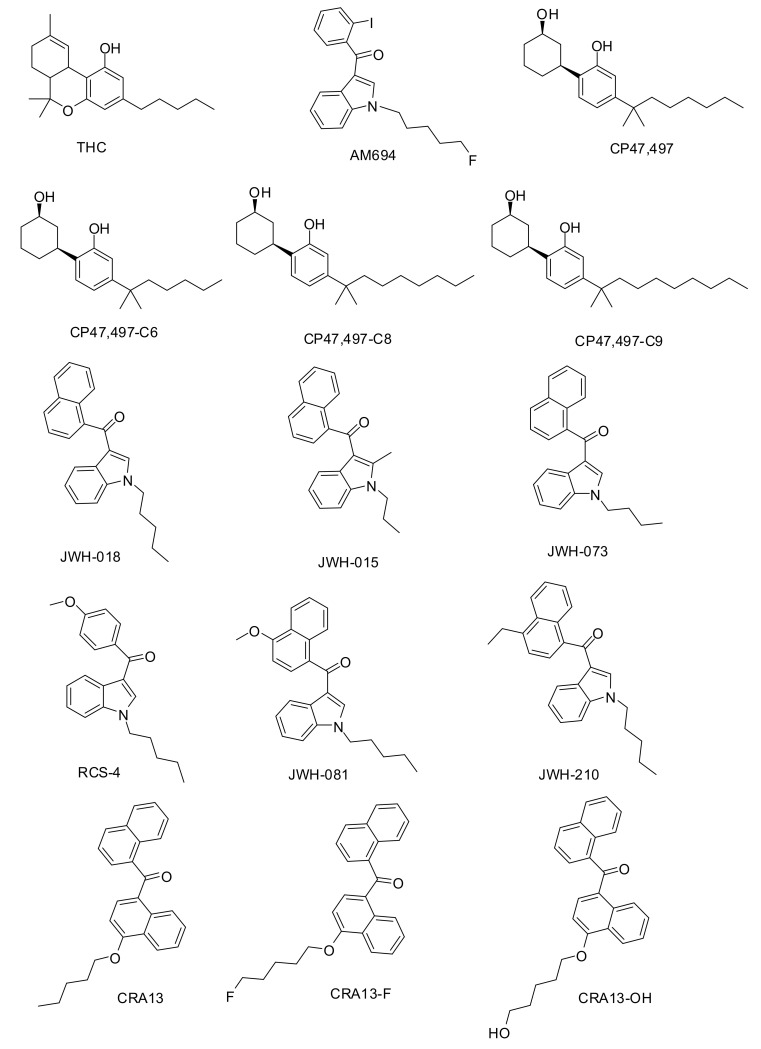
Structure of phytocannabinoid tetrahydrocannabinol (THC) and the synthetic cannabinoids used in this study.

For example, SCs are constituents of widely sold, recreational, designer drug products, usually marketed as herbal incense mixtures named “K2” or “Spice,” which are smoked for their psychoactive effects, including euphoria and hallucination [4]. Serious side effects of SCs, including memory impairments, hypothermic effects, anxiety, and panic, have also been reported [5,6,7,8]. Many studies have demonstrated that cannabinoid receptor 1 (CB1R) mediates the behavioral and psychoactive effects of Δ^9^-tetrahydrocannabinol (THC) and SCs in animals and humans [9,10]. Among SCs, CP47,497 and its homologs (Figure 1) have structural similarities with THC. In vitro studies have shown that CP47,497 binds, with higher affinity than THC, to both the CB1R in the central nervous system and the peripheral CB2R, suggesting that it has the same effects as THC in vivo. Most SCs with psychoactive effects are agonists of CB1R and selectively bind to CB1R with high affinity [11]. Therefore, in vitro CB1R binding assays have been used to predict the abuse potential of SCs at the preliminary screening level [12]. Currently 43 cannabimimetic agents (CB1R agonists) including a variety of SCs are designated as “Schedule 1 substances” controlled by the United States Drug Enforcement Administration [13].

A quantitative structure–activity relationship (QSAR), determined as a regression or classification model, is the relationship between the biological activities of a series of molecules and their structural and physicochemical descriptors. This is one of the major research methods used to predict the biological activities of new drug molecules in the field of rational drug design. Recently, the QSAR method was employed for the assessment of potential hazardous chemicals by government agencies worldwide as a tool to replace expensive and time-consuming animal testing [14]. For example, the U.S. Environmental Protection Agency has established and utilized various QSAR resources to predict and regulate the hazards of new industrial chemicals in the ecological environment as well as in foods and cosmetics.

The binding affinity to CB1R is a validated endpoint associated with the abuse or addiction potential of SCs. Several studies have reported on QSAR modeling of SCs to predict the risks of SC derivatives. However, CB1R binding affinity data for training set compounds has been collected from selected literature reviews or public data sources, which usually contain noisy data [15].

**Figure 2:**
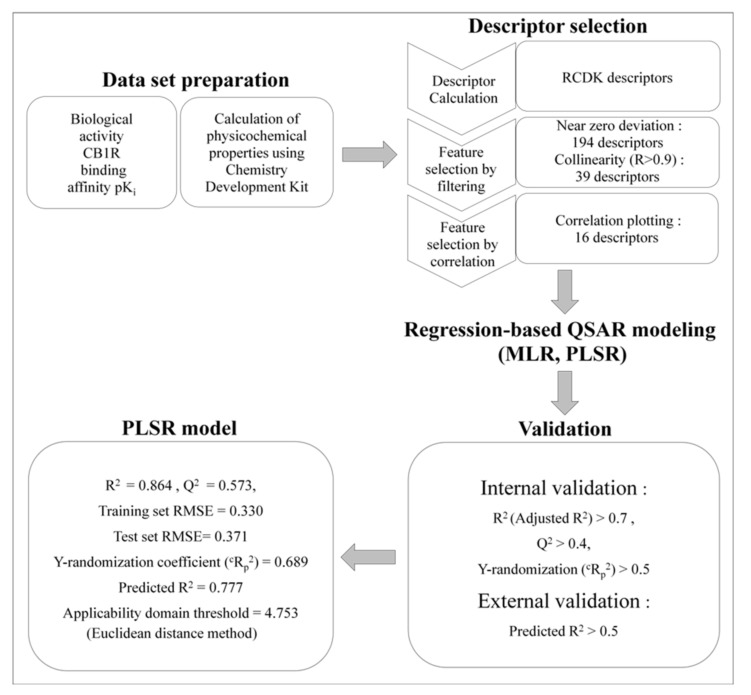
Quantitative structure–activity relationship modeling strategy.

In the present study, we designed a QSAR model of SC derivatives to predict their CB1R-binding affinities using our own experimental results (Figure 2). Regression-based approaches such as multiple linear regression (MLR) and partial least squares regression (PLSR), were used to build numerous QSAR models, and the most reliable PLSR model was selected. Previously, we evaluated the rewarding effects of several SCs using the conditioned place preference (CPP) test [12], which is one of methods used to measure the dependence property of SCs in experimental animals. To evaluate a useful application of our QSAR model for predicting the abuse potential of new SCs, we analyzed the correlation of drug-induced CPP activity with the predicted CB1R-binding affinity values.

## 2. Results and Discussion

### 2.1. QSAR Modeling for Predicting CB1R Binding Affinity of THC and SCs

#### 2.1.1. Dataset Compounds

As shown in Figure 1, the database consists of a natural cannabinoid THC, five naphthoylindoles (JWH-series), two benzoylindoles (AM-694 and RCS-4), three naphthoylnaphthalenes (CRA-series), and four cyclohexylphenols (CP-series). Although the availability of compounds was quite limited, we tried to use diverse SCs as the dataset compounds for QSAR modeling. JWH-series, AM-694, RCS-4, and CP-series are frequently adulterated in commercial herbal incense products, and controlled or outlawed in many countries due to their intoxication and abuse potentials [16].

Cannabis has been known to have therapeutic effects against neurodegenerative disease, cancer, multiple sclerosis, epilepsy, and neuropathic pain. THC is one of the most important ligands responsible for the therapeutic effects of cannabis and a main component of several cannabis-derived medicines. Dronabinol, a commercial name for synthetic THC, was approved for the treatment of anorexia related with AIDS wasting syndrome, and chemotherapy-induced nausea and vomiting in cancer patients [17,18]. Nabiximols (Sativex^TM^), containing a 1:1 ratio of THC:cannabidiol, is also in the market for use as a therapeutic agent to alleviate the spasticity of multiple sclerosis patients [19]. In spite of the clinical beneficial effects of THC, it is associated with psychotropic adverse effect due to the CB1R agonist activity in CNS.

CRA13 is one of naphthoylnaphthalenes, and it is a dual agonist of CB1R and cannabinoid receptor 2 (CB2R). CRA13 was a clinical candidate developed as a peripheral analgesic by the Novartis Pharma company, since it had low blood-brain barrier (BBB) permeability in animal studies [20]. However, when a high dose was administered in human clinical evaluation, it showed the CNS penetration giving adverse effect similar to THC [21]. We synthesized CRA-F and CRA-OH, the analogues of CRA13, to optimize the CB2R selectivity and BBB permeability [22,23]. These CRA-series have not been introduced as illicit drugs yet, but, considering their THC-like property and structural similarity to JWH-series, CRA13 and its derivatives could be distributed illegally in the future. Therefore, we consider these compounds as SC candidates and they were included in the dataset for QSAR modeling tactically. The experimental activity values for dataset compounds are CB1R-binding affinities (K_i_ values) determined using a radio-isotope receptor binding assay.

#### 2.1.2. Feature Selection

**Figure 3:**
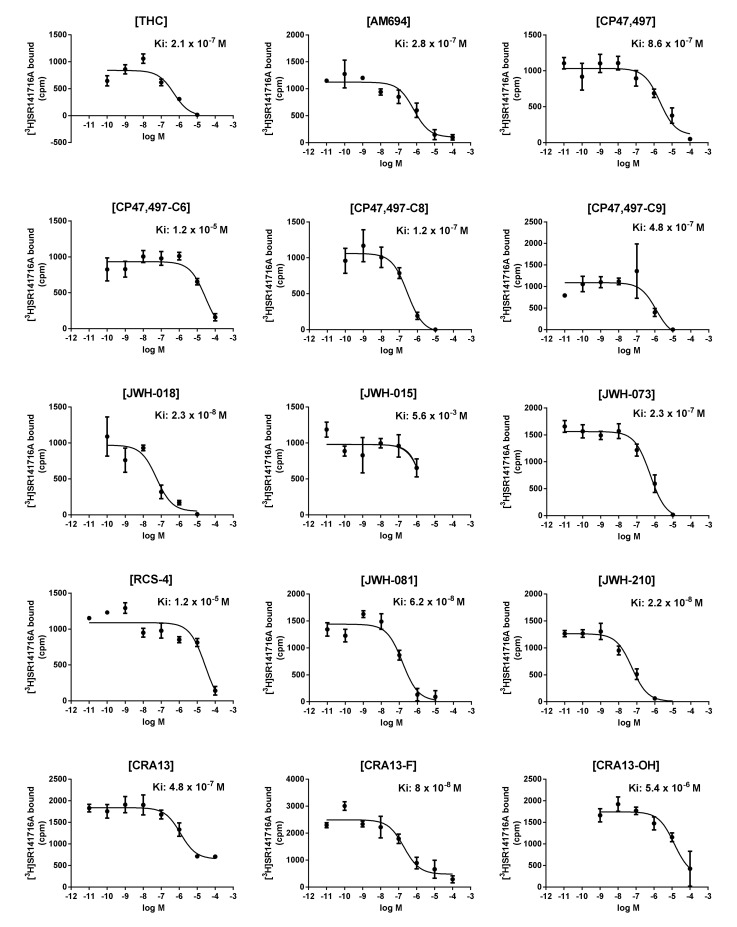
Cannabinoid receptor 1-binding affinities of THC and synthetic cannabinoids.

It is known that CB1R-binding affinity is a reasonable biomarker for the prediction of the abuse or dependence of SCs; herein, we used our experimental CB1R pK_i_ values (Figure 3 and Table S1) as the endpoints of QSAR modeling. To determine dataset compounds, we analyzed pK_i_ values using the *outliers* in the R package [24] and identified the pK_i_ value of JWH-015 as an outlier. After removing JWH-015 from the dataset compounds, compounds with the highest and lowest binding affinities to CB1R were JWH-210 and CP47,497-C6, respectively. The difference in pK_i_ between JWH-210 and CP47,497-C6 was 2.737, which was close to the range of the dependent variable (three in the log scale) that is suitable for building a QSAR model. The compounds were then split into a training set of 11 compounds and a test set of 3 compounds, and used for both MLR and PLSR analyses. Since only 11 SCs were included in the training set to build the model, we employed a sophisticated feature selection procedure (as detailed below) to obtain a reliable QSAR model.

**Figure 4:**
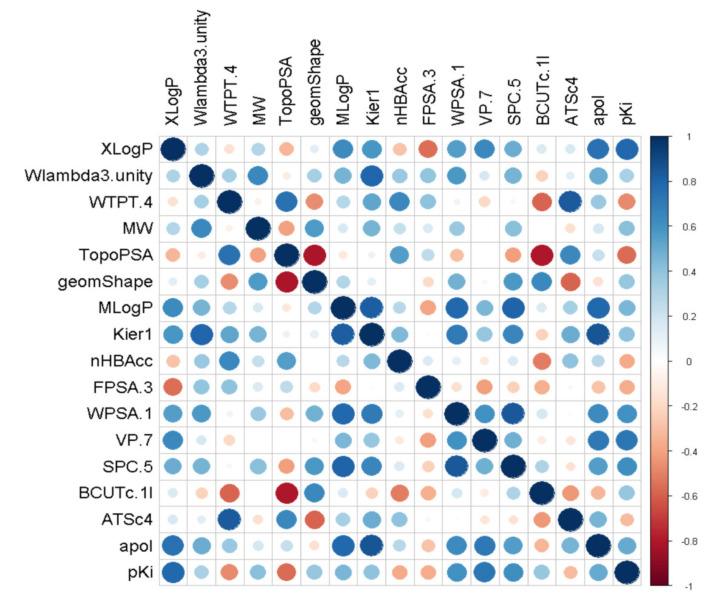
A correlation plot demonstrating the correlation between the dependent variable pK_i_ and descriptor values using different dot sizes and colors. The larger the dot, the stronger the correlation. Blue indicates a positive correlation, and red indicates a negative correlation.

When the descriptors of molecules were calculated by rcdk, the number of features was 286. After removing features with an almost zero variance, 194 features were obtained. Then, additional features that were highly correlated with each other were removed. The cutoff value for correlation was set at 0.9. After excluding highly correlated features, 39 features remained. For these features, we finally selected 16 descriptors (Table S2) that had good correlation with the value of dependent variable CB1R pK_i_ (correlation coefficient > 0.3), using the correlation matrix heat map shown in Figure 4. For MLR, the independent variables were selected by a forward-selection method. The selected independent variables were standardized and applied for QSAR model construction.

#### 2.1.3. MLR Analysis

**Figure 5:**
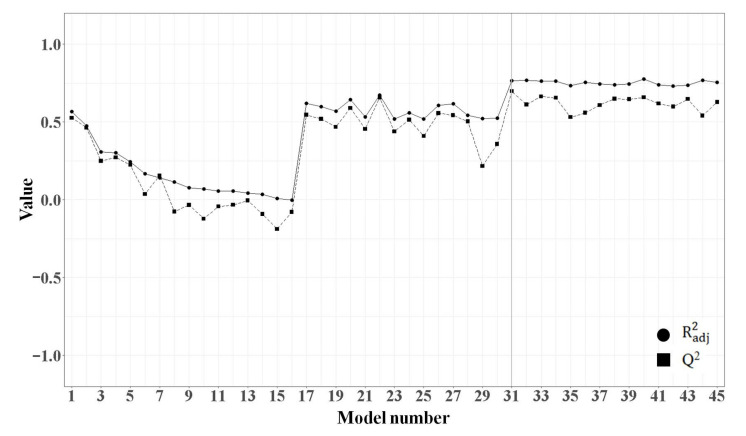
Adjusted R^2^ ($R_{\mathrm{adj}}^{2}$) and Q^2^ values of the multiple linear regression (MLR) models created using the forward selection method. The performance of 45 models during the forward selection process is expressed as a line graph.

**Figure 6:**
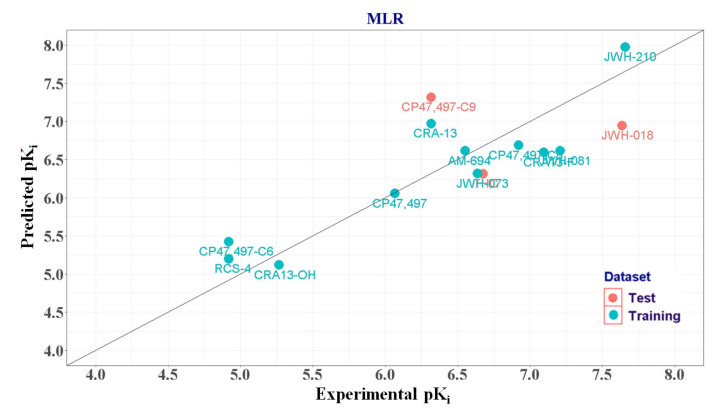
Plot of predicted versus experimental pK_i_ values of multiple linear regression model 31.

Considering the limited size of the training set and the multicollinearity of variables, the MLR model was constructed with a small number of descriptors. As summarized in Table S3 (see the Supplementary Information), we initially built sixteen models using a single descriptor and calculated their adjusted coefficient of determination (adjusted R^2^) values to select the first descriptor to build the MLR model. Model 1 using XLogP (adjusted R^2^ = 0.567) had the highest adjusted R^2^ value. Next, starting from the first descriptor XLogP, further descriptors were included using the forward selection method. Among the models with two descriptor combinations, model 31 with XLogP and ATSc4 had the highest adjusted R^2^ value (0.765). Finally, using up to three descriptors, we established a total of forty-five models. While comparing the adjusted R^2^ and the cross-validated coefficient (Q^2^) values using the plot in Figure 5, we discovered that the adjusted R^2^ reached a statistical plateau after model 31. Therefore, model 31, which was constructed using XLogP and ATSc4, was selected as the most reliable MLR model. To detect the multicollinearity of model 31, we calculated the variance inflation factor (VIF) of each descriptor. The descriptors XLogP and ATSc4 had a low VIF value of 1.029 (much less than 10), thereby indicating the absence of multicollinearity in model 31. Normality of the residuals was confirmed using a Q-Q plot in Figure S1. In Figure 6, the correlation between the actual and predicted CB1R pK_i_ values of the compounds in the training and test sets was plotted. MLR model 31 predicted the CB1R pK_i_ values of the test set compounds with R^2^ = 0.133. The regression equation of MLR model 31 is as follows:

pK_i_ = 0.8038(XLogP) − 0.4269(ATSc4) + 6.3243 (1)

R^2^ = 0.812, F =17.31, *p*-value = 0.001242 (2)

#### 2.1.4. PLSR Analysis

**Figure 7:**
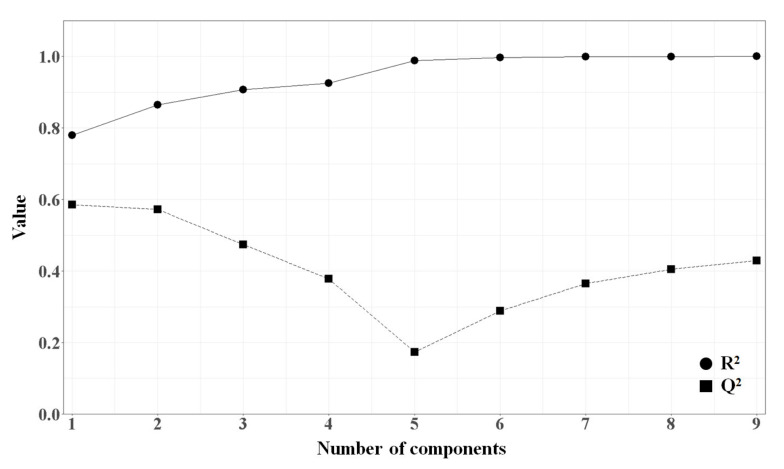
Plot of R^2^ and Q^2^ versus the number of components.

PLSR is known to be more effective when the number of features is much greater than the number of training set samples, because PLSR avoids the problem of collinear features by extracting latent variables. To build PLSR models, 16 descriptors obtained from the feature selection by correlation plotting were used. In order to find the optimal number of principle components, R^2^ and Q^2^ were calculated and plotted against the number of components (Figure 7).

**Figure 8:**
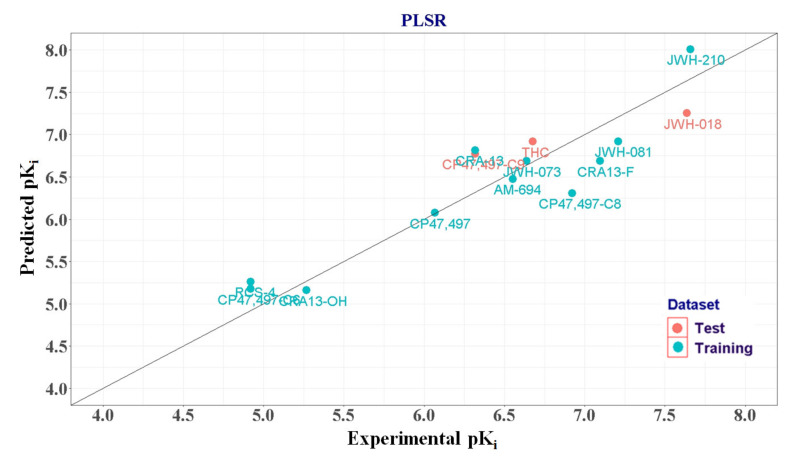
Correlation plot of predicted versus experimental pKi values of the quantitative structure–activity relationship model. PLSR, partial least square regression.

When the first component was used, R^2^ and Q^2^ were 0.780 and 0.585, respectively. By adding a second component, R^2^ was improved to 0.864, Q^2^ was 0.573. When a third component was added, R^2^ was improved to 0.907 but Q^2^ was decreased to 0.475. Therefore, we selected two as the optimal number of components (Figure 7). The correlation between the actual and predicted CB1R pK_i_ values obtained from PLSR is shown in Figure 8, and the normality of the residuals was confirmed using a Q-Q plot shown in Figure S2.

The regression equation of PLSR was as follows:

pK_i_ = 0.1863XLogP + 0.0425Wlambda3.unity − 0.1608WTPT.4+0.0856MW − 0.1178TopoPSA + 0.0224geomShape + 0.0199MLogP + 0.0449Kier1 − 0.1566nHBAcc − 0.0561FPSA.3 + 0.0784WPSA.1 + 0.2057VP.7 + 0.0765SPC.5 + 0.0864BCUTc.1l − 0.0978ATSc4 + 0.0975apol + 6.3243 (3)

R^2^ = 0.864, F =25.41, *p*-value = 0.000342 (4)

#### 2.1.5. Comparison of the Quality of MLR and PLSR Models

As summarized in Table 1, both the MLR and PLSR models were quite stable; however, in the former case, we used only two descriptors for modeling. Thus, the predictability of the MLR model for the test set was relatively low. The variables used in the best MLR model were highly correlated with the pK_i_ values (XLogP = 0.781, and ATSc4 = −0.312,), which is good enough for establishing an MLR model. Therefore, other descriptors, even though they are highly correlated with the pK_i_ values, were left out in the final MLR model mainly due to the multicollinearity problem. However, highly correlated descriptors can be handled in PLSR owing to the orthogonal properties of the eigenvalue; we established PLSR models by using all 16 independent variables using a *pls* algorithm. As expected, the PLSR model had substantially better predictive capabilities than the MLR model. This was especially true for the former regarding the predictability of external test sets, which was significantly higher than that of the latter. Therefore, the PLSR model was chosen as the final QSAR model.

To confirm the robustness of the PLSR model, a Y-randomization test was performed [25]. Twenty-five models were built with randomly scrambled dependent variables and original independent variables and then their R^2^ and Q^2^ values were compared with that of the original PLSR model. The R^2^ and Q^2^ values of all random models were smaller than 0.5, and the calculated Y-randomization coefficient (^c^R_p_^2^) was 0.689 (>0.5), revealing that the PLSR model is reliable and not generated by accidental correlation (Figure S3). The applicability domain (APD) was also defined based on the Euclidean distance method and used to assess the acceptability of external test set compounds. The APD threshold of the PLSR model was determined to be 4.753. The Euclidean distances of the test set compounds (CP47,497-C9, JWH-018, and THC) were within the APD threshold, supporting that their predictive values are reliable (Table 2).

The observed CB1R-binding affinities (pK_i_) of dataset compounds were compared with those predicted by the PLSR QSAR model in Table 2. For all the dataset compounds, our CB1R-binding assay resulted in pK_i_ values ranging from 5.0 to 7.7, which differed by approximately 1 to 2 points from those reported in the literature. The pK_i_ value of the outlier JWH-015 was 2.252, which was considerably far from those of other SCs; thus, it was excluded from the training set.

For CP-47,497 and its homologs in the dataset, the CB1R-binding affinities predicted by the PLSR model increased as the size of the carbon chain attached to the C5 of phenol moiety. This result is similar to the reported structure–activity relationship studies on the various cyclohexylphenol derivatives [26]. CP-47,497 and its homologs (C6, C8, and C9) are under control in many European countries. In addition, CP47,497 and CP47,497-C8 are currently listed as Schedule 1 controlled substances in the United States [27]. Considering the global control status of CP-homologs, we can suggest that a new CP47497 derivative, with a pK_i_ value higher than 6.0 predicted by our PLSR model, should be considered as a candidate for the assessment of abuse potential.

Our experimental pK_i_ values for naphthoylindole derivatives (JWH-type) were in the 6.638–7.658 range, representing the chemical group with the highest CB1R-binding activity (Table 2). Most of the naphthoylindoles identified in commercial incense products are outlawed in many countries including the USA, Germany, and Japan, and the reinforcing effects of several naphthoylindoles (JWH-073, 081, and 210) and THC were investigated by using CPP tests in mice [12]. They all exhibited drug-induced CPP activities, and the order of this activity of naphthoylindoles was JWH-210 > JWH-081 > JWH-073, which is in good agreement with the CB1R-binding affinity order predicted by QSAR. Their predicted pK_i_ values were higher than 6.638. In addition, JWH-018 with predicted pK_i_ = 7.253 induced self-administration (SA) behavior in mice, thereby confirming the rewarding and reinforcing property of the drug [28]. Thereby, we suggest the naphthoylindole-type SCs with the pK_i_ value higher than approximately 6.50 predicted by our QSAR induce CPP or SA behavior in animals demonstrating addictive potentials.

Next, we performed the validation of the PLSR model using an external evaluation set. A set of 62 naphthoylindole cannabinoids (JWH-compounds) was collected from two pieces of the literature to build the database [11,29]. Their experimental K_i_ values of the CB1R were obtained from a radioligand competition assay similar to that used in our study. Among them, 50 compounds were within the applicability domain, and their pK_i_ values were predicted by the PLSR model (Table S4). The Pearson correlation coefficient between the literature value and the predicted value was 0.721 and the predicted R^2^ was 0.702, revealing that the CB1R binding affinity predicted by QSAR is in good correlation with the experimental results of the structure–activity relationship study of JWH-compounds. Interestingly, there are six drugs (JWH-007, −019, −098, −122, −149 and −166), currently listed in the US Schedule I among the dataset compounds, and their predicted pK_i_ values were in the range of 7.085–8.300, higher than 6.50 (Table S4). These results also support that the generated PLSR QSAR model is a reliable tool for the prediction of the CB1R-binding affinity and addiction property of new SCs.

## 3. Materials and Methods

### 3.1. Chemistry

THC and six SCs (AM-694, JWH-015, JWH-073, JWH-081, JWH-210, and RCS-4) were purchased from Cayman Chemical (Ann Arbor, MI, USA). Eight additional SCs, including JWH-018, CRA13 and its derivatives (CRA13-F and CRA13-OH), and CP47,497 and its homologs (CP47,497-C6, C8, and C9) were synthesized. The synthetic methods are briefly described, and the proton nuclear magnetic resonance spectroscopic data of the synthesized compounds are attached in the Supplementary Materials.

### 3.2. In Vitro CB1R-Binding Assay

This test was performed with minor modifications to the previously reported method [29]. ChemiScreen CB1 Cannabinoid Receptor Membrane Preparation (EMD Millipore Corp., Milford, MA, USA) was used.

In saturation binding assays, various concentrations of radioactive [^3^H]-SR141716A (ranging from 0 nM to 20 nM) and a fixed concentration of non-radioactive CP55,940 (7 μM) were incubated with membrane in binding buffer (50 mM Hepes ((4-(2-hydroxyethyl)-1-piperazineethanesulfonic acid)), 5 mM MgCl_2_, 1 mM CaCl_2_, and 0.2% BSA (bovine serum albumin)) for 2 h. The mixture was then transferred to a Whatman® Grade GF/C 96-well filter plate coated with 0.33% polyethyleneimine. After washing the mixture three times using wash buffer (50 mM Hepes, 500 mM NaCl, and 0.1% BSA), the radioactivity noted on the filters was measured using an Ultima Gold liquid scintillation cocktail (PerkinElmer, Waltham, MA, USA). The K_d_ value (6.573 nM) was calculated using the GraphPad Prism 5 software (GraphPad Software, La Jolla, CA, USA).

In competition binding assays, a fixed concentration of [^3^H]-SR141716A (7 nM) and various concentrations of non-radioactive competing ligands (ranging from 10 pM to 100 μM) were incubated with membrane in a binding buffer for 2 h. The mixture was then transferred to a GF/C 96-well filter plate coated with 0.33% polyethyleneimine. After washing the mixture three times using wash buffer, the radioactivity on the filters was measured with an Ultima Gold liquid scintillation cocktail (PerkinElmer, Waltham, MA, USA). The K_i_ values were calculated using the GraphPad Prism 5 software.

The binding displacement curves of competing ligands (ranging from 10 pM to 100 μM) against [^3^H]-SR141716A (7 nM) binding to the CB1 receptor. The data were presented as means ± standard error of means (*n* = 3) (raw data in Table S1).

### 3.3. QSAR Modeling

All QSAR studies were performed by an in-house R script, using the mlr (version 2.17.1), pls (version 2.7-3) package in R program

#### 3.3.1. Preparation of Datasets and Calculation of Molecular Descriptors

The chemical structures of dataset compounds were determined, and their biological activities were assessed using CB1R-binding affinity assay. Additionally, we synthesized 14 compounds for assessing their CB1R-binding Ki values. Eleven of these 14 compounds were included in a training set, and the rest were included in a test set. Furthermore, the dataset was divided into the training (11 compounds) and test (3 compounds) sets, which accounted for 80% and 20%, respectively, of the dataset.

All used compounds were prepared by a sketch module embedded in the Sybyl-X 2.1.1 (Certara Inc., Prinston, NJ, USA). [30] molecular modeling software package in the CentOS Linux 5.4 operating system. Structures of all compounds were determined using sketch modules and saved in mol2 format. All hydrogen atoms and Gasteiger–Hückel charge were added to atoms. To optimize the structures of the compounds, energy minimization was performed until maximum derivatives of 0.001 kcal mol^−1^ Å^−1^ were reached using a standard tripos force field. The data were divided into two sets. One was a training set for the establishment of prediction models, and the other was an external test set for the evaluation of the built prediction models. All dataset compounds (Figure 1) were saved in sdf format.

All molecular descriptors (constitutional, electronic, topological, hybrid, and geometrical) were assessed using the rcdk package [31].

#### 3.3.2. MLR

The primary objective of the MLR was to construct an estimated regression equation (${\hat{y}}_{i}$) by estimating the parent regression equation ($y_{i}$) from the sample. Using the ordinary least squares method, we could estimate the coefficient of the estimated regression equation.

In the following equation, X is denoted by an n × p matrix, where n is the number of observations and p is the number of features. Moreover, Y is denoted by an n × k matrix, where k is the number of dependent variables.

(5) $$y_{i}=\beta_{0}+\beta_{1}x_{i1}+\beta_{2}x_{i2}+\cdots \beta_{p}x_{ip}+\varepsilon_{i}$$

(6) $$\begin{array}{c} \overset{N}{\underset{i=1}{\sum }}e_{i}{}^{2}=\overset{N}{\underset{i=1}{\sum }}{\left(y_{i}-{\hat{y}}_{i}\right)}^{2}\\ \;\hat{\beta }={\left(X^{T}X\right)}^{-1}X^{T}Y \end{array}$$

(7) $${\hat{y}}_{i}={\;\hat{\beta }}_{0}+{\;\hat{\beta }}_{1}x_{i1}+{\;\hat{\beta }}_{2}x_{i2}+{\;\hat{\beta }}_{p}x_{ip}$$

Despite the wide use of MLR, it is inefficient when several variables are included. As there is no variable selection method in MLR, at times, we could not build a model when the number of observations was smaller than the number of variables. To resolve this, we selected descriptors using the forward selection method, and 2 of 16 descriptors were shortlisted. The descriptors were added by comparing the adjusted R^2^ until this value did not increase.

#### 3.3.3. PLSR

MLR is vulnerable to features that are correlated to one another. This is because MLR cannot identify correlated sets that may be more important to the model. To solve this problem, we adopted PLSR method using *pls* packages in R [32].

PLSR is used to analyze or predict a set of dependent variables from a set of independent variables or predictors. It is more useful for handling a large number of correlated and complex features than for handling a limited number of data observations. In the following section, a brief explanation of how PLSR works is outlined.

X is denoted by an n × m matrix, where n is the number of observations and m is the number of features. Moreover, Y is denoted by an n × p matrix, where p is the number of response variables.

Partial least squares analysis (PLS) detects principal components from X that are also relevant for Y. Particularly, PLS explores a set of components that perform a simultaneous decomposition of X and Y with the constraint that these components explain the maximum possible covariance between X and Y. As a result, these components are used to build the model.

$$X=T^{T}P$$

$$Y=U^{T}Q$$

U=BT

T and U are the n × l score vectors that are the projections of X and Y, respectively.

P and Q are the m × l and p × l orthogonal loading vectors, respectively. PLS maximizes the covariance between T and U.

After using the filtering method, it is possible to apply PLSR to the data and find a set of components. Considering the explanation ratio of X and Y, we can choose the number of components to be used in the model. If the number of training sets is 11, two components are sufficient to build QSAR models. Two components explain 77.97% and 8.43% of the training set variance, respectively.

#### 3.3.4. Model Validation

The following statistical parameters were considered to validate QSAR models. To validate the goodness of fit and robustness of the models, we evaluated the R^2^ and Q^2^. In particular, for the MLR model that is affected by the number of descriptors, R^2^ increases as the number of descriptors increases. Therefore, this model was verified using adjusted R^2^ ($R_{\mathrm{adj}}^{2}$). Q^2^ was estimated by the leave-one-out approach. One compound was omitted from the training set, and a new model was built from this slightly smaller training set. Then, using the new model, the activity of the omitted compound was predicted.

R^2^, $R_{\mathrm{adj}}^{2}$, and Q^2^ were calculated using the following equations:

(8) $$R^{2}=1-\frac{\sum {\left(y_{i}-{\hat{y}}_{i}\right)}^{2}}{\sum {\left(y_{i}-\bar{y}\right)}^{2}}$$

(9) $$R_{\mathrm{adj}}^{2}=1-\frac{\left(1-R^{2}\right)\left(N-1\right)}{N-p-1}$$

(10) $$Q^{2}=1-\frac{\sum {\left(y_{i}-\hat{y}\right)}^{2}}{\sum {\left(y_{i}-\bar{y}\right)}^{2}}$$

where $y_{i}\;\mathrm{and}\;{\hat{y}}_{i}$ are the actual and predicted activities of the *i*th training set, $\bar{y}$ is the average activity of the training set, N represents the number of training sets, and p represents the number of descriptors.

To evaluate the predictability of the model, we used the predicted R^2^ ($R_{\mathrm{pred}}^{2}$) value which is calculated with test set data.

(11) $$R_{\mathrm{pred}}^{2}=1-\frac{\sum {\left(y_{i}-\hat{y}\right)}^{2}}{\sum \left(y_{i}-\bar{y}\right)}$$

where $y_{i}\;\mathrm{and}\;{\hat{y}}_{i}$ are the actual and predicted activities of the *i*th test set, and $\bar{y}$ is the average of the training set response variable. Furthermore, we validated the normal distribution of residuals by Q-Q plotting. Q-Q is a plot of quintiles from each dataset. If the Q-Q points are on a straight line with a 45-degree slope, the data can be interpreted to follow normal distribution.

A Y-randomization test was performed to verify that the QSAR model was not constructed by an accidental correlation between the dependent variable and the descriptor. The Y-randomization test was repeated 25 times, and the Y-randomization coefficient ^c^R_p_^2^ was calculated as follow to validate robustness of model:

$${}^{c}R_{p^{2}}=\;R\;\times \sqrt{R^{2}-R_{r}^{2}}$$

R is correlation coefficient for original model and Rr is average R of random models. If the ^c^R_p_^2^ is greater than 0.5, the model passes the test.

#### 3.3.5. Applicability Domain of the Model

Euclidean distances were used to define the applicability domain of the QSAR models based on the descriptor space of training set. The Euclidean distance of the test set was calculated with the compound which has the smallest distance in the training set and compared to the defined applicability domain (APD) threshold [33]. Euclidean distance and APD were calculated as follows:

$$\mathrm{Euclidean}\mathrm{distance}\;=\;d(x,\;y)\;=\;\sqrt{\sum {\left(x_{i}-y_{i}\right)}^{2}}$$

APD = <d> + Zσ

Among all the Euclidean distances of the training set, we selected a distance lower than the average. We defined the average and standard deviation of this set as <d> and σ. Z is the cutoff value and it was decided as 0.5. If the Euclidean distance of the test set is less than the defined APD threshold, the predicted value is reliable.

#### 3.3.6. Validation of PLSR Model Using an External Evaluation Set

We prepared the database containing 62 compounds obtained from the literature [11,29]. The Euclidean distance was calculated for each molecule to select the compounds which entered into the APD (threshold = 4.753). As listed in Table S4, 50 compounds were within the APD and used as a validation set. The correlation between the experimental value and the predicted value was calculated by Pearson correlation coefficient.

Pearson correlation coefficient is the test statistic that measures the statistical relationship and is calculated as follows:

$$r_{xy}\;=\;\frac{\sum \left(X_{i}-\;\bar{X}\right)\left(Y_{i}-\;\bar{Y}\right)}{\sqrt{\sum {\left(X_{i}-\;\bar{X}\right)}^{2}}\sqrt{\sum {\left(Y_{i}-\;\bar{Y}\right)}^{2}}}$$

where $\bar{X,}\;\bar{Y,}$ is mean of variables X and Y, respectively.

## 4. Conclusions

Considering the prevalence of SCs and their harmful effects, we need a reliable tool to predict the abuse potential of new SC congeners. This study aimed to build QSAR models, which could predict the CB1R-binding affinity of SCs. We conducted QSAR modeling of SCs using two regression methods (PLSR and MLR) using our own CB1R-binding assay results as training data. We obtained a PLSR model with good statistical performance with a limited number of data observations. As a result, we suggest boundary pK_i_ values for the CB1R binding of SCs that may result in dependence or abuse. The resulting QSAR model can be used to predict the CB1R-binding affinity and suggest a further validation of in vivo addictive potentials (CPP and SA behavior) of drugs, correlating with the predicted CB1R-binding affinity. The current study provided not only a novel strategy for QSAR modeling but also an efficient tool to predict the abuse or addiction potential of SCs for the purpose of controlling their illicit use.

## Figures and Tables

**Table 1:** Statistical parameters of multiple linear regression (MLR) and partial least squares regression (PLSR).

Model	R^2^	Adjusted R^2^ (R^2^_adj_)	Predicted R^2^(R_pred_^2^)	Training Set ^1^ RMSE	Test Se tRMSE	Q^2^
MLR model 31	0.812	0.765	0.133	0.387	0.732	0.698
PLSR	0.864	-	0.777	0.330	0.371	0.573

^1^ RMSE, root mean square error.

**Table 2:** Cannabinoid receptor 1-binding affinity (pKi) of synthetic cannabinoids predicted by the partial least squares (PLSR) quantitative structure–activity relationship model.

	Compound Name	Observed pK_i_	PLSR			In Vivo Rewarding Responses
			Predicted pK_i_	^1^ Residual	^2^ APD	
Training set	AM-694	6.553	6.474	0.079		
	CRA-13	6.319	6.816	−0.497		
	CP47,497-C6	4.921	5.174	−0.253		
	CP47,497	6.066	6.075	−0.009		
	CP47,497-C8	6.921	6.308	0.613		
	CRA13-F	7.097	6.687	0.41		
	CRA13-OH	5.268	5.160	0.108		
	RCS-4	4.921	5.26	−0.339		
	JWH-073	6.638	6.688	−0.05		^3^ CPP [12]
	JWH-081	7.208	6.92	0.288		CPP [12]
	JWH-210	7.658	8.007	−0.349		CPP [12]
Test set	CP47,497-C9	6.319	6.773	−0.454	1.903	
	JWH-018	7.638	7.253	0.385	2.887	^4^ SA [17]
	THC	6.678	6.917	−0.239	3.917	CPP [12]

^1^ Residual: difference between the observed and predicted pK_i_ values. ^2^ APD: applicability domain (threshold = 4.753). ^3^ CPP: conditioned place preference. ^4^ SA, self-administration.

## Supplementary Materials

The following are available online. Synthesis and characterization of synthetic cannabinoids, Table S1: CB1R-binding affinity raw data for 15 synthetic cannabinoids, Table S2: List of descriptors used for QSAR models, Table S3: Statistical analysis of MLR models, Figure S1: Q-Q plots of the residuals from MLR model 31 showing a normal distribution, Figure S2: Q-Q plots of the residuals from PLSR model showing a normal distribution, Figure S3: Y-Randomization analysis of the generated PLSR model, Table S4: CB1R-binding affinity (pK_i_) of JWH-series compounds predicted by the PLSR model.
