# Supplementary material for: QSAR Model for Predicting the Cannabinoid Receptor 1 Binding Affinity and Dependence Potential of Synthetic Cannabinoids
Source: Molecules. 2020 Dec 21;25(24):6057. doi: 10.3390/molecules25246057 (PMC7767513; doi:10.3390/molecules25246057)
Supplement: Supplementary file 1 [file molecules-25-06057-s001.pdf]

SUPPLEMENTARY INFORMATION

QSAR Model for Predicting the Cannabinoid Receptor 1 Binding Affinity and Dependence Potential of Synthetic Cannabinoids

Wonyoung Lee[1], So-Jung Park[1], Ji-Young Hwang[1], Kwang-Hyun Hur[1], Yong Sup Lee[2], Jongmin Kim[3], Xiaodi Zhao[1], Aekyung Park[4], Kyung Hoon Min[3], Choon-Gon Jang [1,*] and Hyun-Ju Park [1,*]

[1] School of Pharmacy, Sungkyunkwan University, Suwon, Gyeonggi-do 16419, Republic of Korea; wonyoung1007@naver.com (W.L.); sojung1024@gmail.com (S.-J. P.); innetjy@hotmail.com (J.-Y.H.); khh508@naver.com (K.-H. H.); zhaoxiaodi1019@gmail.com (X.Z.)

[2] Department of Pharmacy, College of Pharmacy, Kyung Hee University, Seoul 02447, Republic of Korea; kyslee@khu.ac.kr

[3] College of Pharmacy, Chung-Ang University, Seoul, 06974, Republic of Korea; drugfriend2@gmail.com (J.K.); khmin@cau.ac.kr (K.H.M.)

[4] College of Pharmacy, Sunchon National University, Suncheon 57922, Korea; parkak11@scnu.ac.kr

* Correspondences: jang@skku.edu; Tel.: +82-31-2907780 (C.-G. J.); hyunju85@skku.edu; Tel.: + 82-31- 290 7719 (H.-J. P.)

Syntheses of Synthetic Cannabinoids

Synthesis of CRA13, CRA13-F, and CRA13-OH

The derivatives of CRA13, CRA13-F [1] and CRA13-OH [2] were synthesized as published previously.

CRA13 was synthesized starting from 1-naphthol and 1-naphthoyl chloride in 3-steps sequence of reactions as described previously [3] and its structure and purity were confirmed by the following spectroscopic and HPLC analyses.

^{1}H NMR (400 MHz, CDCl3) δ 9.00 (d, J = 8.3 Hz, 1H), 8.39 (dd, J = 8.3 Hz, 0.9 Hz, 1H), 8.23 (dd, J = 8.3 Hz, 0.9 Hz, 1H), 7.97 (d, J = 8.3 Hz, 1H), 7.90 (dd, J = 7.8 Hz, 1.5 Hz, 1H), 7.66 (ddd, J = 8.31 Hz, 6.83 Hz, 1.48 Hz, 1H), 7.59–7.42 (m, 6H), 6.61(J = 8.3 Hz, 1H), 4.13 (t, J = 5.4 Hz, 2H), 1.97–1.88 (m, 2H), 1.59–1.49 (m, 2H), 1.47–1.37 (m, 2H), 0.97 (t, J = 7.3 Hz, 3H); 13C NMR (100 MHz, CDCl3) δ 199.1, 159.2, 139.1, 135.5, 134.0, 133.1, 131.5, 131.3, 129.0, 128.6, 128.5, 128.3, 127.5, 126.6, 126.3, 126.2 (2C), 126.2, 124.7, 122.7, 103.0, 68.8, 29.0, 28.6, 22.7, 14.3; HR-MS calcd for C26H25O2 [M+H]+ 369.1849, found 369.1863; HPLC purity = 99.06%, Luna (C18) 5 μm (150 x 4.6 mm), mobile phase: 0.1% formic acid in water(A)/acetonitrile(B), 0–30 min (A/B = 10/90).

(a) ^{1}H-NMR Spectrum of CRA13 (400 MHz, CDCl3)

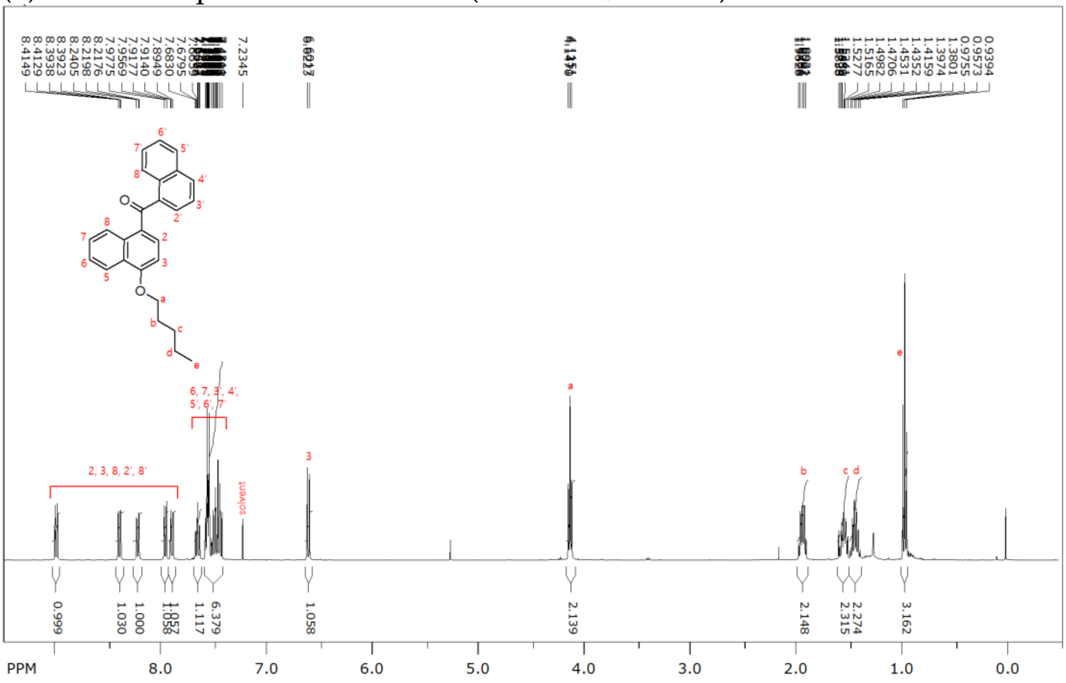

(b) ^{13}C-NMR spectrum of CRA13 (100 MHz, CDCl3)

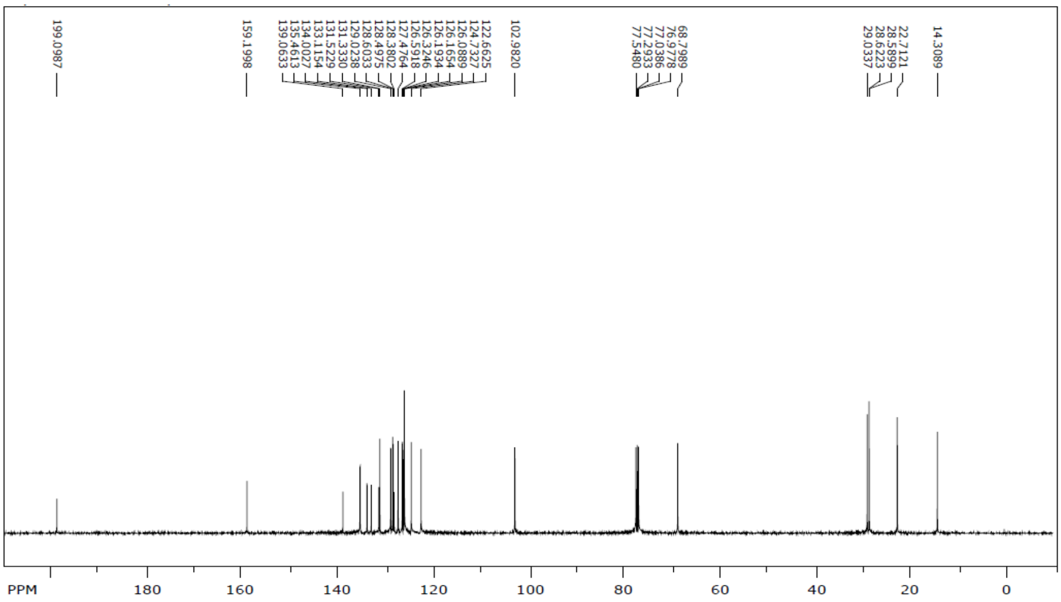

(c) High Resolution Mass spectrum of CRA13.

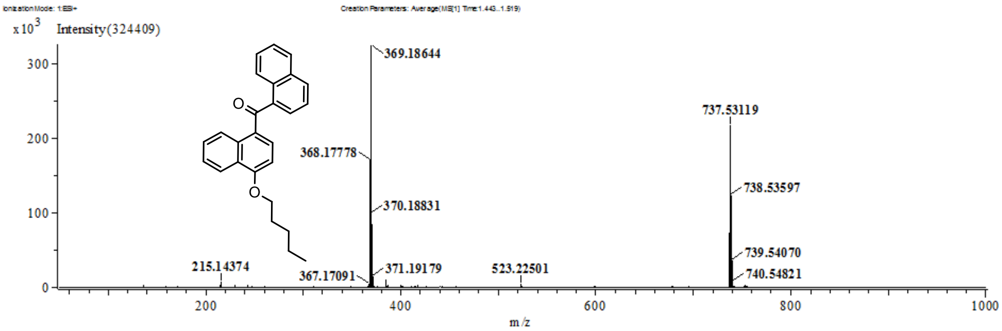

(d) High Performance Liquid Chromatography (HPLC) chromatogram of CRA13.

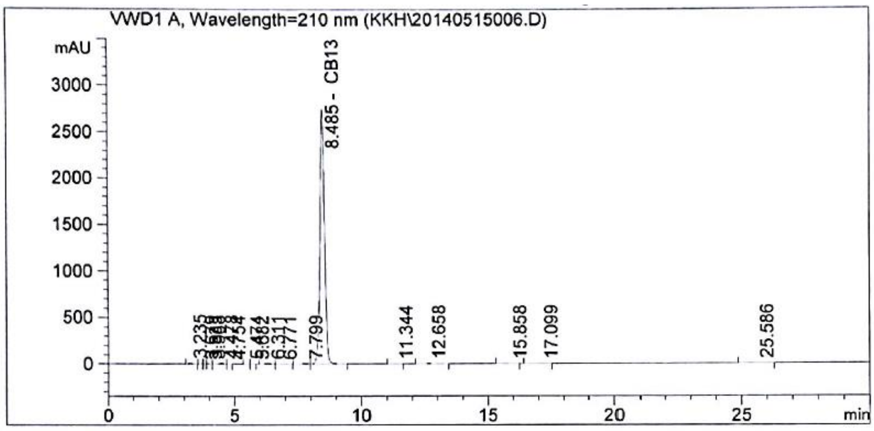

HPLC Condition

Column	Conditions	Detector	Sample

Luna (C18) 5 ㎛ 150 x 4.6 mm	Mobile Phase A: 0.1% formic acid in water<br>Mobile Phase B: 0.1% formic acid in acetonitrile<br>0–30 min; A/B = 10/90	210 nm	Concentration :<br>5 ㎎/㎖ in acetonitrile;<br>5 ㎕ injection

Results: 99.06% purity

Peak #	RetTime [min]	Type	Width [min]	Area [mAU*s]	Area %	Name
1	3.235	BB	0.0834	63.13132	0.1802	?
2	3.679	BV	0.0902	4.26878e-1	1.218e-3	?
3	3.835	VV	0.0778	2.30263	6.571e-3	?
4	3.968	VB	0.0946	9.23103	0.0263	?
5	4.478	BV	0.1196	58.67265	0.1674	?
6	4.754	VBA	0.0972	2.81685	8.039e-3	?
7	5.474	BV	0.1117	6.31314	0.0180	?
8	5.682	VB	0.1058	1.80776	5.159e-3	?
9	6.311	BV	0.1482	52.76944	0.1506	?
10	6.771	VB	0.1943	8.98942	0.0257	?
11	7.799	BV	0.1867	47.05533	0.1343	?
12	8.485	VB	0.1939	3.47119e4	99.0618	CB13
13	11.344	BB	0.2269	12.12432	0.0346	?
14	12.658	BB	0.3155	29.82164	0.0851	?
15	15.858	BB	0.3055	5.08271	0.0145	?
16	17.099	BB	0.3294	16.73203	0.0478	?
17	25.586	BB	0.3974	11.48984	0.0328	?

Synthesis of JWH-018

JWH-018 was synthesized by alkylation of indole with pentyl bromide followed by acylation of the resulting 1-pentylindole with 1-naphthoyl chloride as described [4].

(a)^{1}H-NMR spectrum of JWH-018.

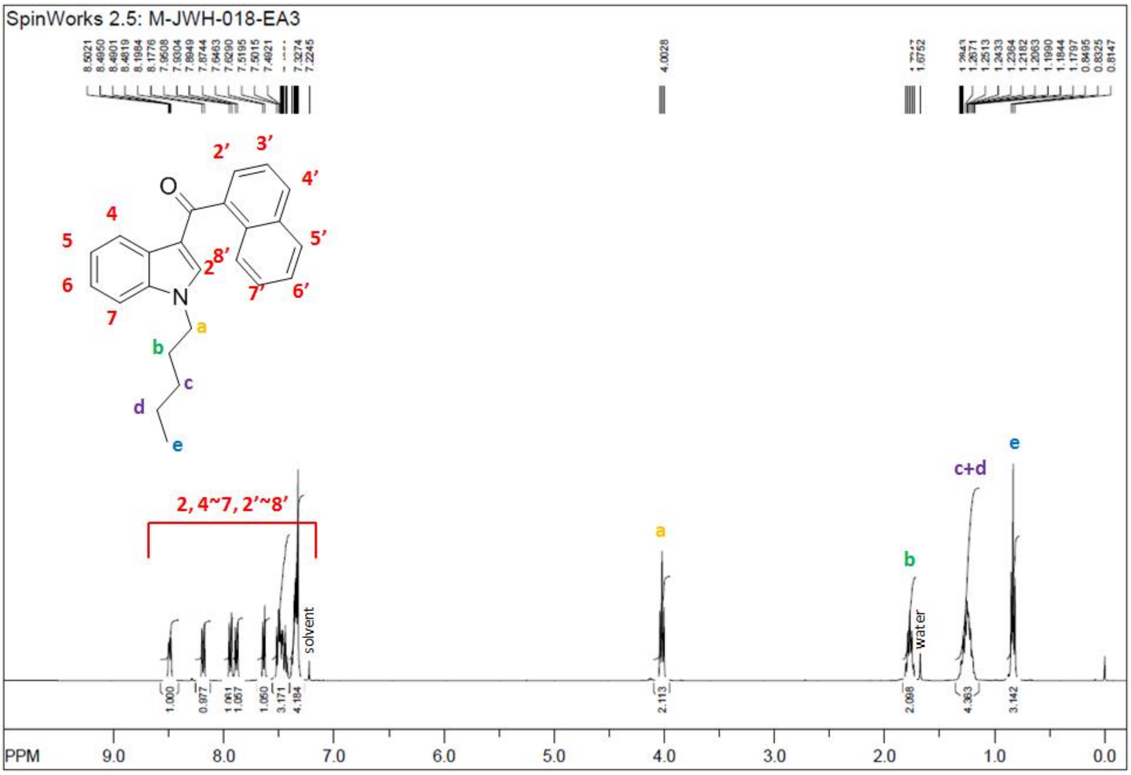

(b) ^{13}C-NMR spectrum of JWH-018.

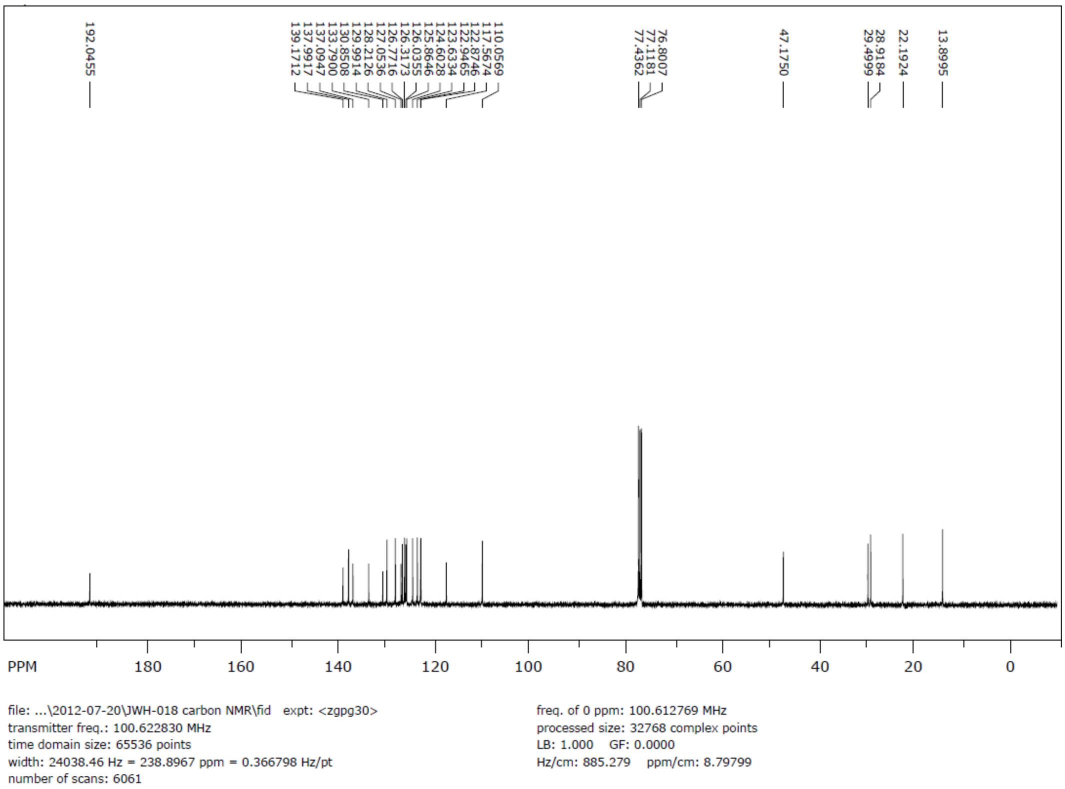

(c) High Resolution Mass spectrum of JWH-018.

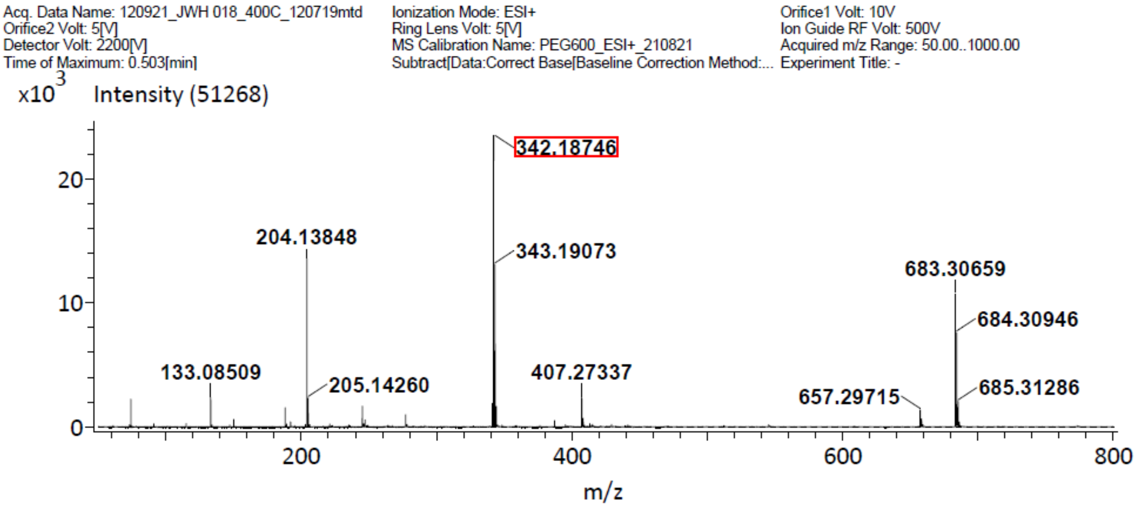

Synthesis of CP47,497 and its homologs (CP47,497-C6, C8, and C9)

CP47,497 and its homologs (**CP47,497-C6, C8, and C9**) were synthesized by the following procedure. The derivatives of 1-methoxy CP47,497 were prepared

according to the reported method [5]. Treatment of 1-methoxy CP47,497 derivatives with 1-propanthiol and n-butyllithium in HMPA gave the desired compounds (CP47,497-C6, C8, and C9).

2-((1S,3R)-3-hydroxycyclohexyl)-5-(2-methyloctan-2-yl)phenol (CP47,497)
^{1}H NMR (300 MHz, $CDCl_3$) δ 7.09 (d, J = 8.4 Hz, 1H), 6.87 (dd, J = 8.4 Hz, 2.1 Hz, 1H), 6.70 (d, J = 2.1 Hz, 1H), 3.69-3.86 (m, 1H), 2.79-2.99 (m, 1H), 2.13-2.27 (m, 2H), 1.98-2.13 (m, 2H), 1.80-1.98 (m, 2H), 1.49-1.75 (m, 4H), 1.26 (s, 6H), 1.01-1.30 (m, 8H), 0.79-0.94 (m, 3H) LRMS(EI) m/z 317.9

2-((1S,3R)-3-hydroxycyclohexyl)-5-(2-methylheptan-2-yl)phenol (CP47,497-C6)
1H NMR (300 MHz, CDCl3) : δ 7.08 (d, J = 7.8 Hz, 1H), 6.86 (dd, J = 7.8 Hz, 2.1 Hz, 1H), 6.70 (d, J = 2.1 Hz, 1H), 3.71-3.90 (m, 1H), 2.80-3.00 (m, 1H), 2.11-2.40 (m, 2H), 1.95-2.10 (m, 2H), 1.78-1.94 (m, 2H), 1.30-1.74 (m, 4H), 1.25 (s, 6H), 0.98-1.40 (m, 6H), 0.80-0.90 (m, 3H) ; HRMS(ESI) m/z calcd for C20H32O2 [M+1]+ : 304.2402, found 305.2479

2-((1S,3R)-3-hydroxycyclohexyl)-5-(2-methylnonan-2-yl)phenol (CP47,497-C8)
1H NMR (300 MHz, CDCl3) : δ 7.08 (d, J = 8.1 Hz, 1H), 6.86 (dd, J = 8.1 Hz, 1.8 Hz, 1H), 6.69 (d, J = 1.8 Hz, 1H), 3.70-3.83 (m, 1H), 2.81-2.94 (m, 1H), 2.12-2.41 (m, 2H), 1.95-2.13 (m, 2H), 1.78-1.96 (m, 2H), 1.37-1.63 (m, 4H), 1.25 (s, 6H), 0.98-1.38 (m, 10H), 0.80-0.98 (m, 3H) ; HRMS(ESI) m/z calcd for C22H36O2 [M+1]+ : 332.2715, found 333.2787

2-((1S,3R)-3-hydroxycyclohexyl)-5-(2-methyldecan-2-yl)phenol (CP47,497-C9)
1H NMR (300 MHz, CDCl3) : δ 7.08 (d, J = 8.1 Hz, 1H), 6.86 (dd, J = 8.1 Hz, 1.8 Hz, 1H), 6.69 (d, J = 1.8 Hz, 1H), 3.70-3.84 (m, 1H), 2.81-2.94 (m, 1H), 1.78-2.3 (m, 6H), 1.36-1.60 (m, 6H), 1.24 (s, 6H), 0.97-1.36 (m, 12H), 0.86 (t, J = 6.6 Hz, 3H) ; HRMS(ESI) m/z calcd for C23H38O2 [M+1]+ : 346.2872, found 347.2943

Table S1. CB1R-binding affinity raw data for 15 synthetic cannabinoids.

	THC			AM694			CP47,497			CP47,497-C6			CP47,497-C8			CP47,497-C9			JWH-018			JWH-015		
M	1			2			3			4			5			6			7			8		
10^{-4}				169	0	124	71	-2	83	60	217	203												
10^{-5}	49	0	0	337	55	56	293	250	588	650	579	736	0	0	0	0	0	0	0	0	24			
10^{-6}	356	287	285	831	367	599	716	572	771	917	1018	1100	268	95	208	316	587	294	121	158	230	408	816	737
10^{-7}	514	616	724	1098	740	717	1106	755	826	791	1108	1039	920	775	662	553	2601	916	130	394	433	656	1162	1060
10^{-8}	981	961	1235	868	1056	897	923	1235	1164	848	1136	1031	1058	1225	739	1129	1249	979	972	966	854	899	968	1122
10^{-9}	700	891	991	1184	1254	1172	875	1118	1317	868	623	998	1176	1554	776	1070	1338	896	615	572	1095	676	499	1312
10^{-10}	532	570	837	1765	1174	884	552	1031	1173	738	604	1136	1033	1215	626	1159	1303	708	784	853	1634	987	919	755
10^{-11}				1169	1131	1148	1237	962	1118							791	831	748				1190	1365	1006
Ki(M)	2.06E-07			2.83E-07			8.62E-07			1.18E-05			1.15E-07			4.81E-07			2.26E-08			0.005558		
	JWH-073			RCS-4			JWH-081			JWH-210			CRA13			CRA13-F			CRA13-OH					
M	9			10			11			12			13			14			15					
10^{-4}				202	23	199							649	702	762	57	299	499	1025	-347	597			
10^{-5}	43	-27	35	702	871	868	-143	245	170	-39	35	-3	729	761	644	454	205	1313	1067	1038	1363			
10^{-6}	785	728	267	877	909	770	-19	358	60	-10	107	91	1189	1643	1165	1321	686	659	1327	1777	1321			
10^{-7}	1429	1049	1179	801	971	1164	747	1041	810	319	571	646	1872	1516	1656	1540	1702	2137	1747	1929	1631			
10^{-8}	1763	1647	1301	918	1068	861	1380	1776	1311	1018	1049	790	2211	2054	1450	1494	2873	2297	1651	1886	2226			
10^{-9}	1414	1640	1417	1154	1323	1403	1606	1532	1748	1006	1475	1435	1880	2255	1602	2122	2491	2402	1514	1504	1972			
10^{-10}	1670	1706	1320	1203	1203	1288	1218	1433	1030	1164	1403	1241	1462	2009	1796	3161	3175	2689						
10^{-11}	1733	1797	1443	1174	1100	1187	1255	1588	1188	1162	1368	1269	1936	1656	1899	2118	2352	2418						
Ki(M)	2.28E-07			1.16E-05			6.16E-08			2.20E-08			4.80E-07			8.00E-08			5.42E-06					

Table S2. List of descriptors used for QSAR models.

Descriptor Name	Descriptor Type	Descriptor class	Definition
XLogP	XLogP	Constitutional Descriptor	Prediction of logP based on the atom-type method called XLogP
VP.7	ChiPath	Topological Descriptor	Evaluates chi path descriptors Valence path orders 7
SPC.5	ChiPathCluster	Topological Descriptor	Evaluates chi path cluster descriptors, Simple path cluster, order 5
TopoPSA	TPSA	Topological Descriptor	Calculation of topological polar surface area based on fragment contributions (TPSA)
WTPT.4	WeightedPath	Topological Descriptor	Evaluates the weighted path descriptors.sum of path lengths starting from oxygens
Kier1	KappaShapeIndices	Topological Descriptor	Kier and Hall kappa molecular shape indices compare the molecular graph with minimal and maximal molecular graphs, First kappa shape index
MW	Weight	Constitutional Descriptor	Molecular weight

BCUTc.1l	BCUT	Hybrid descriptor	Eigenvalue based descriptor noted for its utility in chemical diversity, nhigh lowest partial charge weighted BCUTS(nhigh : The number of highest eigenvalue)
MLogP	MannholdLogP	Constitutional descriptor	Prediction of logP based on the number of carbon and hetero atoms.
Wlambda3.unity	WHIM	Hybrid descriptor	Holistic descriptors described by Todeschini et al (Todeschini, R. and Gramatica, P.. Persepectives in Drug Discovery and Design. 1998. null). Wlambda3 directional WHIM descriptors.
WPSA.1	CPSA	Electronic & geometrical descriptor	Calculates Charged Partial Surface Area (CPSA)<br>sum of surface area on positive parts of molecule * total molecular surface area / 1000
FPSA.3	CPSA	Electronic & geometrical descriptor	Calculates Charged Partial Surface Area (CPSA)<br>Charge weighted partial positive surface area/ total molecular surface area
apol	APolDescriptor	Electronic descriptor	Sum of the atomic polarizabilities<br>(including implicit hydrogens).
geomShape	PetitjeanShapeIndex	Topological descriptor	Evaluates the Petitjean shape indices,<br>(geometric shape index)
nHBAcc	HBondAcceptorCount	Electronic descriptor	This descriptor calculates the number of hydrogen bond acceptors using a slightly simplified version of the PHACIR atom types.
ATSc4	AutocorrelationDescriptorCharge	Topological descriptor	Calculates the Autocorrelation of a Topological Structure autocorrelation descriptor, where the weight equal to the charges.

Table S3. Statistical Analysis of MLR models.

MLR Model number	Descriptor	R^2	Adjusted R^2_{adj}	Predicted R^2_{pred}	training set RMSE	test set RMSE	Q^2
1	XLogP	0.611	0.567	-0.857	0.558	1.071	0.527
2	VP.7	0.528	0.475	-0.776	0.614	1.047	0.463
3	SPC.5	0.376	0.307	0.008	0.706	0.783	0.25
4	WPSA.1	0.372	0.302	0.216	0.708	0.695	0.272
5	TopoPSA	0.319	0.244	0.539	0.737	0.533	0.224
6	apol	0.252	0.168	-0.755	0.773	1.041	0.037
7	WTPT.4	0.227	0.141	0.546	0.786	0.529	0.156
8	MLogP	0.203	0.114	0.117	0.798	0.738	-0.075
9	MW	0.17	0.078	-0.477	0.814	0.955	-0.033
10	Kier1	0.163	0.07	-0.793	0.818	1.052	-0.121
11	BCUTc.1l	0.152	0.058	0.799	0.823	0.352	-0.043
12	geomShape	0.15	0.056	-0.3	0.824	0.896	-0.032
13	nHBAcc	0.14	0.044	-0.247	0.829	0.877	-0.005
14	FPSA.3	0.132	0.035	0.556	0.833	0.524	-0.092
15	Wlambda3.unity	0.107	0.008	-0.983	0.844	1.106	-0.188
16	ATSc4	0.097	-0.003	0.466	0.849	0.574	-0.078
17	VP.7, XLogP	0.696	0.621	-0.607	0.492	0.996	0.546
18	SPC.5, XLogP	0.679	0.599	0.097	0.506	0.746	0.52
19	WPSA.1, XLogP	0.655	0.569	-0.334	0.525	0.907	0.468
20	TopoPSA, XLogP	0.714	0.643	0.098	0.478	0.746	0.59
21	apol, XLogP	0.625	0.532	-0.635	0.547	1.005	0.455
22	WTPT.4, XLogP	0.738	0.672	-0.378	0.458	0.922	0.656
23	MLogP, XLogP	0.615	0.518	-0.965	0.555	1.101	0.439
24	MW, XLogP	0.647	0.559	-0.986	0.531	1.107	0.515
25	Kier1, XLogP	0.616	0.519	-0.718	0.554	1.03	0.41
26	BCUTc.1l, XLogP	0.686	0.607	0.049	0.501	0.766	0.556
27	geomShape, XLogP	0.694	0.618	-0.374	0.494	0.921	0.544
28	nHBAcc, XLogP	0.636	0.544	-0.402	0.539	0.93	0.503
29	FPSA.3, XLogP	0.618	0.522	-1.123	0.553	1.145	0.217
30	Wlambda3.unity, XLogP	0.619	0.524	-1.23	0.551	1.173	0.358
**31**	**ATSc4, XLogP**	**0.812**	**0.765**	**0.133**	**0.387**	**0.732**	**0.698**
32	VP.7, ATSc4, XLogP	0.838	0.768	0.04	0.36	0.77	0.613
33	SPC.5, ATSc4, XLogP	0.834	0.762	0.465	0.364	0.575	0.664
34	WPSA.1, ATSc4, XLogP	0.834	0.762	0.42	0.365	0.598	0.655
35	TopoPSA, ATSc4, XLogP	0.813	0.733	0.083	0.387	0.752	0.531
36	apol, ATSc4, XLogP	0.829	0.756	0.039	0.369	0.77	0.559
37	WTPT.4, ATSc4, XLogP	0.82	0.743	0.13	0.379	0.733	0.609
38	MLogP, ATSc4, XLogP	0.818	0.74	0.269	0.382	0.672	0.649
39	MW, ATSc4, XLogP	0.821	0.744	0.098	0.378	0.746	0.646
40	Kier1, ATSc4, XLogP	0.844	0.777	0.09	0.353	0.75	0.658
41	BCUTc.1l, ATSc4, XLogP	0.817	0.739	0.28	0.382	0.667	0.618
42	geomShape, ATSc4, XLogP	0.813	0.732	0.157	0.387	0.721	0.599
43	nHBAcc, ATSc4, XLogP	0.816	0.737	0.029	0.383	0.774	0.648
44	FPSA.3, ATSc4, XLogP	0.838	0.769	-0.158	0.359	0.846	0.54
45	Wlambda3.unity, ATSc4, XLogP	0.829	0.756	-0.153	0.369	0.843	0.628

(R^2: coefficient of determination data set, R^2_{adj} : adjusted R^2, R^2_{pred} : R^2 for external test set, RMSE: root mean squared Error, Q^2: cross-validated coefficient of determination (Leave-One-Out method))

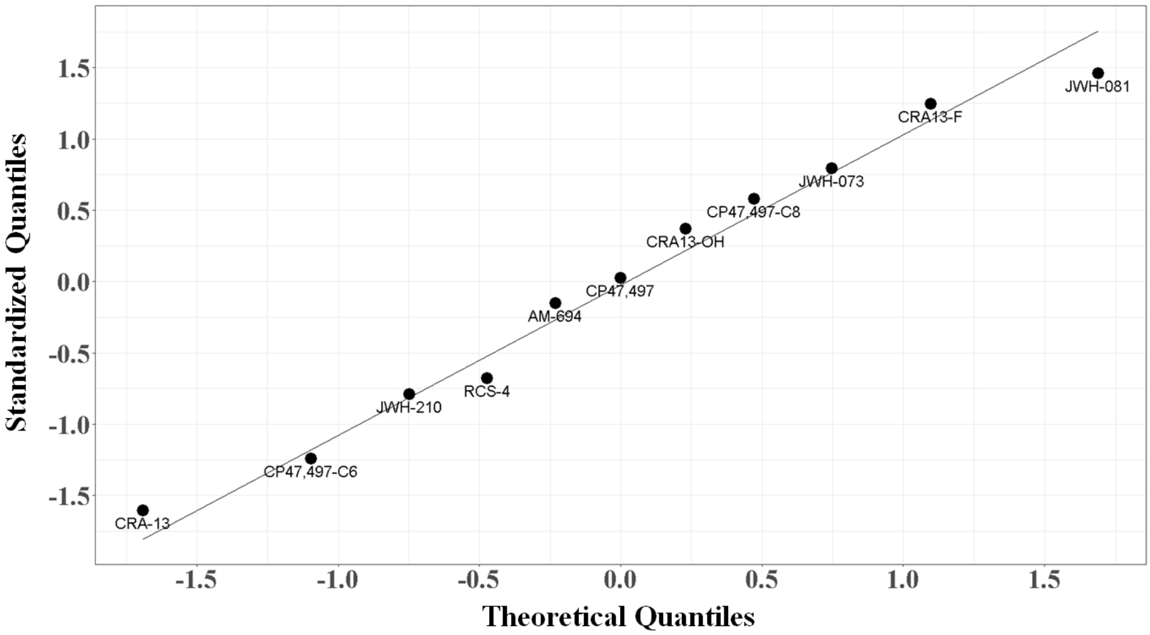

Figure S1. Q-Q plot of the residuals from MLR model 31 showing a normal distribution.

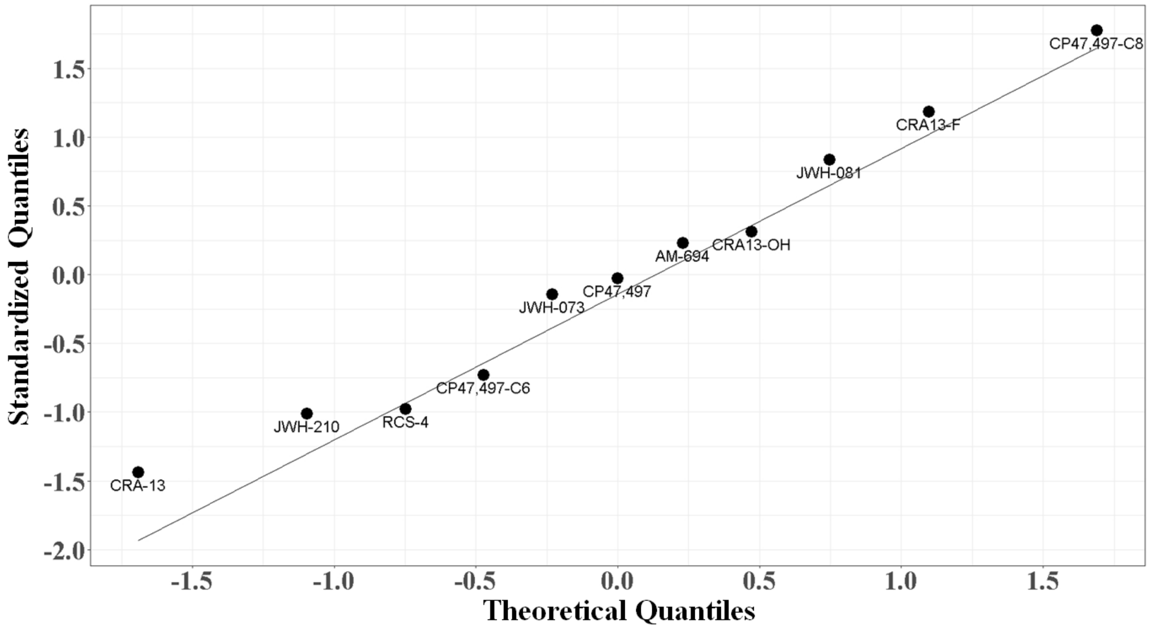

Figure S2. Q-Q plot of the residuals from PLSR model showing a normal distribution.

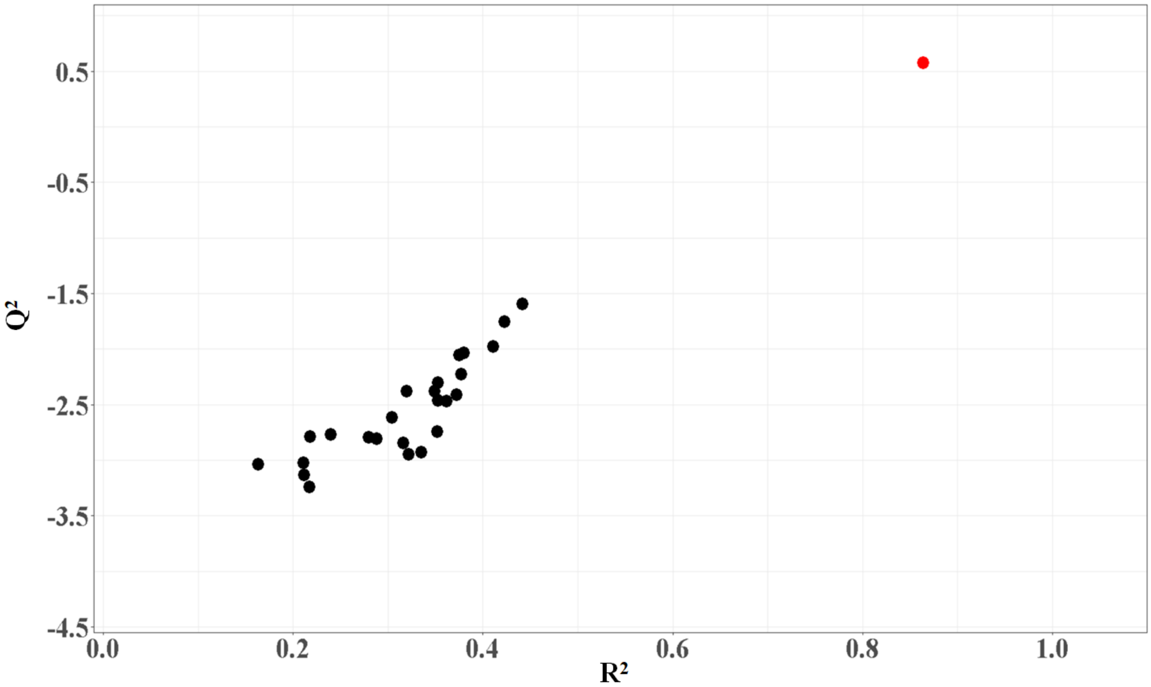

Figure S3. Y-Randomization analysis of the generated PLSR model. Black dots indicate Y-randomization models, and a red dot indicates the PLSR model.

Table S4. CB1R-binding affinity (pK_i) of JWH-series compounds predicted by the PLSR model.

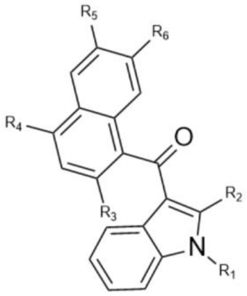

Compound Name	R_1	R_2	R_3	R_4	R_5	R_6	pK_i value of literature	Predicted pK_i	[1]Residual	Eclidean Distance (APD= 4.753)
*JWH-007	C_5H_{11}	CH_3	H	H	H	H	8.022	**7.490**	0.532	2.036
JWH-015	C_3H_7	CH_3	H	H	H	H	6.785	6.436	0.349	3.540
JWH-016	C_4H_9	CH_3	H	H	H	H	7.658	7.323	0.335	3.186
*JWH-019	C_6H_{13}	H	H	H	H	H	8.009	**7.962**	0.047	4.280
JWH-043	C_2H_5	CH_3	H	H	H	H	5.928	6.049	-0.121	4.233
JWH-046	C_3H_7	CH_3	H	H	H	CH_3	6.465	6.750	-0.285	3.762
JWH-047	C_4H_9	CH_3	H	H	H	CH_3	7.231	7.349	-0.118	2.930
JWH-048	C_5H_{11}	CH_3	H	H	H	CH_3	7.971	7.924	0.047	2.231

JWH-049	C_6H_{13}	CH_3	H	H	H	CH_3	7.259	8.679	-1.420	4.466
JWH-071	C_2H_5	H	H	H	H	H	5.873	5.594	0.279	4.171
JWH-072	C_3H_7	H	H	H	H	H	5.979	6.167	-0.188	2.057
JWH-076	C_3H_7	H	H	H	H	CH_3	6.67	6.471	0.199	2.099
JWH-078	C_2H_5	H	H	OCH_3	H	H	6.088	5.595	0.493	4.180
JWH-079	C_3H_7	H	H	OCH_3	H	H	7.201	6.211	0.990	3.933
JWH-080	C_4H_9	H	H	OCH_3	H	H	8.252	6.528	1.724	2.163
JWH-082	C_6H_{13}	H	H	OCH_3	H	H	8.276	7.689	0.587	4.130
JWH-094	C_3H_7	CH_3	H	OCH_3	H	H	6.322	6.294	0.028	3.614
JWH-096	C_4H_9	CH_3	H	OCH_3	H	H	7.472	7.344	0.128	2.852
*JWH-098	C_5H_{11}	CH_3	H	OCH_3	H	H	8.347	**8.300**	0.047	2.864
JWH-120	C_3H_7	H	H	CH_3	H	H	5.977	6.398	-0.421	3.388
*JWH-122	C_5H_{11}	H	H	CH_3	H	H	9.161	**7.966**	1.195	4.015
JWH-148	C_3H_7	CH_3	H	CH_3	H	H	6.91	6.620	0.290	4.447
JWH-149	C_5H_{11}	CH_3	H	CH_3	H	H	8.301	7.778	0.523	2.802
JWH-153	C_5H_{11}	CH_3	H	H	OCH_3	H	6.602	7.614	-1.012	3.537
JWH-159	C_5H_{11}	CH_3	H	H	H	OCH_3	7.347	7.433	-0.086	2.755
JWH-160	C_3H_7	CH_3	H	H	H	OCH_3	5.805	6.242	-0.437	3.744
JWH-163	C_3H_7	H	H	H	OCH_3	H	5.627	5.977	-0.350	3.440
JWH-164	C_5H_{11}	H	H	H	H	OCH_3	8.18	7.565	0.615	1.360
JWH-165	C_3H_7	H	H	H	H	OCH_3	6.69	6.222	0.468	3.965
*JWH-166	C_5H_{11}	H	H	H	OCH_3	H	7.357	**7.085**	0.272	1.677
JWH-180	C_3H_7	H	H	C_3H_7	H	H	7.585	7.519	0.066	3.776
JWH-181	C_5H_{11}	CH_3	H	C_3H_7	H	H	8.886	8.971	-0.085	3.569
JWH-182	C_5H_{11}	H	H	C_3H_7	H	H	9.187	9.191	-0.004	2.851
JWH-189	C_3H_7	CH_3	H	C_3H_7	H	H	7.284	7.753	-0.469	3.236
JWH-211	C_3H_7	CH_3	H	C_2H_5	H	H	7.155	7.295	-0.140	3.935
JWH-212	C_3H_7	H	H	C_2H_5	H	H	7.481	7.223	0.258	2.837
JWH-213	C_5H_{11}	CH_3	H	C_2H_5	H	H	8.824	8.408	0.416	2.396
JWH-234	C_5H_{11}	H	H	H	H	C_2H_5	8.076	8.077	-0.001	2.626
JWH-235	C_3H_7	H	H	H	H	C_2H_5	6.471	6.998	-0.527	2.291
JWH-236	C_3H_7	CH_3	H	H	H	C_2H_5	5.869	7.282	-1.413	4.504
JWH-239	C_3H_7	H	H	C_4H_9	H	H	6.466	8.009	-1.543	3.599
JWH-240	C_5H_{11}	H	H	C_4H_9	H	H	7.854	9.132	-1.278	4.054
JWH-241	C_3H_7	CH_3	H	C_4H_9	H	H	6.833	8.301	-1.468	2.507
JWH-258	C_5H_{11}	H	H	OC_2H_5	H	H	8.337	8.234	0.103	2.784
JWH-259	C_3H_7	H	H	OC_2H_5	H	H	6.658	6.423	0.235	2.435
JWH-260	C_5H_{11}	CH_3	H	OC_2H_5	H	H	7.538	8.028	-0.490	4.556

JWH-261	C_3H_7	CH_3	H	OC_2H_5	H	H	6.115	6.796	-0.681	2.617
JWH-262	C_5H_{11}	CH_3	H	H	H	C_2H_5	7.553	8.485	-0.932	3.254
JWH-267	C_5H_{11}	H	OCH_3	H	H	H	6.419	7.256	-0.837	3.240
JWH-268	C_5H_{11}	CH_3	OCH_3	H	H	H	5.86	7.838	-1.978	4.255

(* Compound currently in US schedule I; [1]Residual: difference between the observed and predicted pK_i values.)

References

[1] Hassan, A.H., Park, K.T., Kim, H.J., Lee, H.J., Kwon, Y.H., Hwang, J.Y., et al. Fluorinated CRA13 analogues: Synthesis, in vitro evaluation, radiosynthesis, in silico and in vivo PET study. Bioorganic Chemistry. 2020, 103834.

[2] Hassan, A.H., Cho, M.C., Kim, H.I., Yang, J.S., Park, K.T., Hwang, J.Y., et al. Synthesis of oxidative metabolites of CRA13 and their analogs: Identification of CRA13 active metabolites and analogs thereof with selective CB2R affinity. Bioorganic & medicinal chemistry. 2018, 26, 5069-78.

[3] Dziadulewicz, E.K., Bevan, S.J., Brain, C.T., Coote, P.R., Culshaw, A.J., Davis, A.J., et al. Naphthalen-1-yl-(4-pentyloxynaphthalen-1-yl) methanone: a potent, orally bioavailable human CB1/CB2 dual agonist with antihyperalgesic properties and restricted central nervous system penetration. Journal of medicinal chemistry. 2007, 50, 3851-6.

[4] Appendino, G., Minassi, A., Taglialatela-Scafati, O. Recreational drug discovery: natural products as lead structures for the synthesis of smart drugs. Natural product reports. 2014, 31, 880-904.

[5] Huffman, J.W., Hepburn, S.A., Reggio, P.H., Hurst, D.P., Wiley, J.L., Martin, B.R. Synthesis and pharmacology of 1-methoxy analogs of CP-47,497. Bioorganic & medicinal chemistry. 2010, 18, 5475-82.
